# A Review on Additive Manufacturing of Functional Gradient Piezoceramic

**DOI:** 10.3390/mi13071129

**Published:** 2022-07-17

**Authors:** Anton Sotov, Artem Kantyukov, Anatoliy Popovich, Vadim Sufiiarov

**Affiliations:** Institute of Mechanical Engineering, Materials, and Transport, Peter the Great St. Petersburg Polytechnic University, 195251 St. Petersburg, Russia; kantyukov.artem@mail.ru (A.K.); director@immet.spbstu.ru (A.P.); vadim.spbstu@yandex.ru (V.S.)

**Keywords:** additive manufacturing, functionally graded materials, functionally graded piezoceramic, gradient chemical composition, controlled gradient porosity, disordered gradient porosity

## Abstract

Functionally graded piezoceramics are a new generation of engineering materials whose final properties are determined by a chemical composition gradient (volume distribution), material microstructure, or design characteristics. This review analyzes possible ways to create a functionally graded piezoceramic material (gradient chemical composition, gradient porosity—controlled and disordered porosity) by additive manufacturing methods, to control such materials’ functional characteristics. An analysis of the creation of gradient piezoceramics using binder jetting technology is presented in more detail. The review shows that today, the creation of functional gradient piezoceramics by additive manufacturing is a poorly-studied but promising research area, due to the rapid development of the additive manufacturing market and their unique features in shaping parts.

## 1. Introduction

Graded piezoceramic materials are functionally graded materials (FGMs). FGMs are a new generation of technical materials, the final properties of which are determined by a given gradient (volume distribution) of the chemical composition, material microstructure, or structural characteristics during design. This feature allows FGMs to achieve material properties that are impossible to obtain using homogeneous alloys and composites and allows distributing properties in areas where needed. Initially, FGMs were classified as conventional composite materials, depending on the constituent components [1]. There are various constituent components for the production of FGMs, such as metal-to-metal [2,3,4,5,6,7], metal-to-ceramic [8,9,10,11,12], ceramic-to-ceramic [13,14,15,16,17,18,19,20], or ceramic-to-polymer [21,22,23,24,25,26]. Today, due to the development of new technologies and new FGMs application areas, their classification is greatly expanding. It is worth marking six generally accepted criteria for FGMs classification [27]:

1. According to the state during FGMs processing (solid-state, liquid-state, and deposition processes).
2. According to FGMs structure (continuous and discontinuous graded material).
3. According to the type of FGMs gradient (composition, microstructure, and porosity).
4. According to the nature of FGMs gradation process (constructive and transport processing).
5. According to the FGMs scale and dimensions (thin FGMs, thick/bulk FGMs).
6. According to FGMs application (biomaterial, aerospace, automotive, defense, cutting tools, nuclear reactor, smart structure, turbine blades, and sports equipment).

Over the past two decades, advances in additive manufacturing (AM) have proven that gradient design is technologically and economically feasible, especially for prototypes and small-scale production [28,29,30,31], even with components that are not entirely compatible or homogeneous. AM suggests additional benefits of precision and repeatability to achieve desired gradients and properties [32,33,34,35,36]. AM of piezoceramic has been discussed in various perspectives, based on materials and applications [37,38,39,40,41,42,43,44,45,46,47].

The functionally graded piezoceramic (FGP) is the most abundant material for energy harvesting, where flexible and elastic devices are used to convert mechanical energy into electrical energy sensors in, for example: use as insulators with spatially varying dielectric permittivity and high dielectric anisotropy, which is highly relevant for aircraft radio equipment manufacturing within the aviation industry; in the manufacture of transceiver modules for hydroacoustic antennas within the maritime industry; and in the manufacture of sensors for steam-water path pressure monitoring in the nuclear industry. The FGP is also used in transformation optics for electromagnetic radiation controlling; in actuators for improved reliability and creating higher-order movements; and in resonator elements for fabricating structures with electromagnetic properties of metamaterials and adjustable operating frequencies. Another important application is biomedicine, namely wearable biomedical diagnostic devices, and bone regenerative therapies, which improve implant ingrowth and long-term stability.

In this work, our main attention is focused on analyzing possible ways of creating and controlling the functional characteristics of graded piezoceramic materials with various types of FGMs manufactured by AM. This paper provides a systematic and comprehensive analysis of FGP. The organization of this paper is as follows: Section 1 presents a brief introduction to FGMs. The review of FGP and its classification are described in Section 2. Section 3 shows the review of 3D printing FGP by binder jetting (BJ) technology. Section 4 gives our conclusions and remarks.

## 2. Functionally Graded Piezoceramic

Currently, the development and improvement of new piezoceramic materials are mainly carried out by traditional manufacturing. The most common methods are: pressing, spark plasma sintering, cold isostatic pressing, tape casting, gel casting, injection molding, and others [48,49,50,51,52,53,54]. The experience gained in traditional manufacturing suggests that the material’s structure and properties are determined not only by its composition, but also by the impact of the selected shaping and sintering processes. Parts made by traditional methods achieve high piezoelectric properties; however, the geometric shape and texturing capabilities limit them. Obtaining functional gradient piezoceramics (FGP) by AM is a new direction in smart materials manufacturing. Piezoceramic is a smart material that converts electrical energy into a mechanical one, or vice versa. Since this material is widely used in various technology fields, such as the electronics, military, medical and automotive industries, the issue of improving functional properties is relevant.

Applying the FGMs concept for piezoceramics makes it possible to increase the efficiency of these materials. For example, while harvesting an energy using FGMs piezocomposites, the maximum performance was achieved by using an approach based on optimizing discrete materials. Accordingly, the main investigated parameters are the polarization direction of the piezoelectric material, and the gradation material. Vatanabe et al. [55] compared FGMs piezocomposites with their analogs without material gradation. The microscopic stresses in FGMs piezocomposites were significantly lower than in similar materials without gradation. Thus, creating a graded material in piezocomposites has led to the accumulation of more electricity. Rubio et al. [56] used the topology optimization approach to study the effect of creating a gradient material on the functional properties of piezoceramic transducers. Achieving the maximum natural frequency of the eigenmode was chosen as a functional parameter. The gradation in the thickness direction was significantly more efficient for natural frequencies, than the gradation in the longitudinal direction.

Apart from piezoceramic components, the FGP is used to produce parts from dielectric materials. Isakov et al. [57] demonstrated 3D printing of the FGP as a dielectric material with spatially varying dielectric permittivity, and high dielectric anisotropy. The studied FGP samples were with a gradient chemical composition. They were fabricated by the fused deposition modeling (FDM) method, using filaments filled with micro-ceramic powder with controlled permittivity and losses. The obtained results allowed investigators to change the microwave operating frequency, and the magnitude of Mie-type resonances, to get characteristics similar to a metamaterial.

Castles et al. [58] demonstrated the results of microwave dielectric properties 3D-printed from a ceramic/polymer composite with high dielectric constants. A series of composites were fabricated with different barium titanate (BT) microparticles content in acrylonitrile butadiene styrene (ABS) polymer matrix. Microwave dielectric properties of 3D-printed samples with BT content up to 70 wt.% were studied using a 15 GHz split post dielectric resonator. The relative permittivity was 2.6–8.7, and the loss tangents were in the range of 0.005–0.027. Permittivities were reproducible over the entire process and matched those of unprinted bulk materials to within ~1%. The received results show that 3D printing is promising for dielectric composites manufacturing.

The FGP is also used in transformation optics for electromagnetic radiation controlling. Polymers have excellent processability to produce filaments, thin films, sheets, bulk materials, and three-dimensional shapes. Various types of fillers should be added to be useful for transformation optics, and the range of resulting materials is rapidly expanding [59]. For example, common bulk polymers, such as polyethylene (PE) and polypropylene (PP), are readily processed but provide only dielectric permittivity in the range of 2–3 at approximately 10 GHz, limiting their use in transformation optics devices. On the other hand, ceramics such as BT are brittle and much less versatile in processing; their volumetric permittivity at microwave frequencies can be several orders of magnitude greater than that of polymers. Therefore, by dispersing BT microparticles into a polymer matrix and creating FGMs with a variable chemical composition, it is possible to obtain processed materials with a permittivity up to 20 in the frequency range of 12–18 GHz.

**Figure 1:**
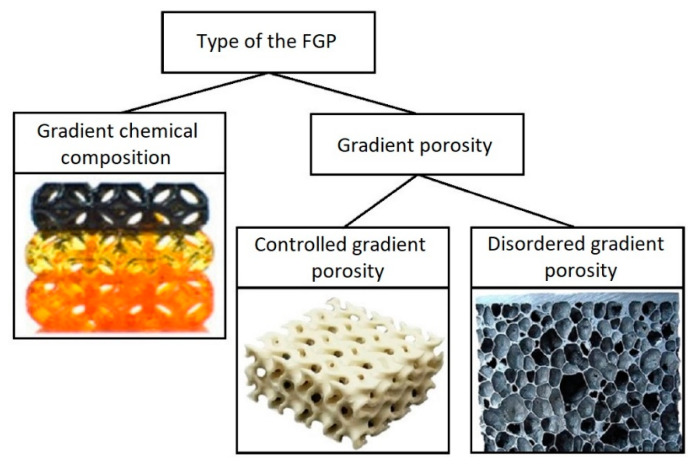
FGP classification by gradient type.

Based on literature analysis in the field of producing FGMs by AM, it has been found that final functional material properties are determined mainly by the gradient type. In addition to the general classification presented above, the introduction of an additional criterion according to the type of FGP has been proposed; namely, the “porosity” gradient type is further divided into two criteria: “controlled porosity” and “disordered porosity” (Figure 1).

The following is an analysis of possible ways to create and control the functional characteristics of a gradient piezoceramic material, using various types of FGMs gradient obtained by AM; namely, the type of gradient chemical composition or multi-material 3D printing, and gradient porosity—controlled and disordered porosity.

### 2.1. Type of the FGP

#### 2.1.1. Gradient Chemical Composition of the FGP (Multi-Material 3D Printing)

The use of FGMs with a gradient chemical composition has found wide application in materials used to eliminate thermal stresses and fractures [60], increase fracture toughness [61], and also increase wear resistance [62]. This conception was further extended to smart materials, such as piezoceramics, to improve reliability and create higher-order movements in actuators [63,64,65]. General research focuses on manufacturing a monolithic FGP by doping additional chemical elements and compounds to influence the change in resistivity, conductivity, piezoelectric coefficient, permittivity, porosity, or a combination of these properties [66,67,68,69,70]. The nature of a monolithic FGP implies the manifestation of the required material properties throughout the entire thickness of the sample, where energy can be spent on a less active material, thereby reducing the performance and efficiency of the actuator.

Paul W. Alexander et al. [71] used a type of gradation in chemical composition to change piezoelectric coefficients and permittivity by concentrating the applied electric field in the material with a large piezoelectric effect. This approach made it possible to increase the drive deviation and reduce the values of control electrical potentials. The authors studied various materials and ratios to create FGP with the required functional properties. The authors also demonstrated the dependence graphs of the influence of material variations on the piezoelectric properties of an actuator. The samples contained a piezoceramic of lead zirconate titanate (PZT), with a high piezoelectric coefficient, and BT with a high dielectric constant. The FGP samples were printed using micro-fabrication by co-extrusion (MFCX) process. The authors noted that the MFCX process allows for precise spatial control of material variations to produce more complex gradient profiles in one, two, and three dimensions. The composition of the samples ranged from pure PZT to 77/23 vol.% PZT/BT. To study possible variations of FGP materials, the authors tested four samples for each material composition, to determine the effect on the relative permittivity and piezoelectric coefficients. Results showed the nonlinear rise in permittivity from 4022 for pure PZT, to a plateau of approximately 9642 at 20 vol.% BT. The 20 vol.% change in material composition accounts for a 140% increase in the dielectric properties. In addition, the tests show a reduction of 96% in piezoelectric activity across the tested composition range, with the dielectric coefficient dropping from 283 pm/V to 6 pm/V. It was established that when the resulting piezoelectric strain gradient is coupled with the variation in permittivity, the two gradients work synergistically to increase the deformations and electric efficiency of the FGP actuator.

**Figure 2:**
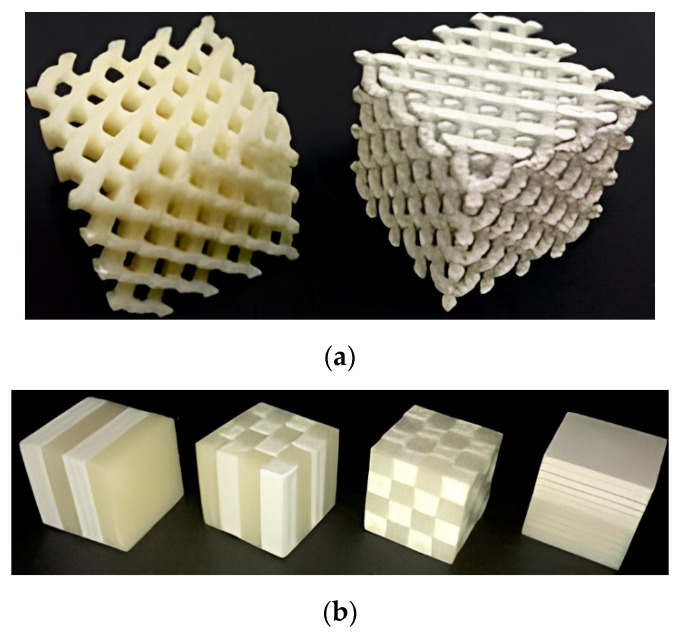
(**a**) 3D-printed sample from ABS polymer (left, ε′ = 2.57) and 50 wt.% BT/ABS polymer composite (right, ε′ = 4.95). Scale: each cubic structure has an overall side length of 32 mm (8 mm unit cell); (**b**) 3D-printed samples with gradient chemical composition using a combination of ABS polymer and 50 wt.% BT in ABS polymer composite. Scale: each cubic structure has a side length of 16 mm (available under Creative Commons license [58]).

Castles et al. [58] used multi-material 3D printing for manufacturing FGP. They demonstrated the results of a study in to the microwave dielectric properties of printed ceramic/polymer composite parts with high dielectric constants. The starting material was BT powder with an average particle size of 3 μm, dispersed in an ABS polymer matrix. This material was extruded into filaments suitable for a commercial desktop FDM 3D printer (Makerbot Replicator 2X, Makerbot Industries, New York, NY, USA), which belongs to the material extrusion category. The FGP samples were printed using standard settings: e.g., 230 °C nozzle temperature and 110 °C printed bed temperature. The authors produced eight filaments with BT powder content from 0 to 70 wt.% in increments of 10 wt.%. The filament became brittle and unsuitable for 3D printing at a higher powder content. Figure 2 shows FGP samples with a gradient chemical composition.

**Figure 3:**
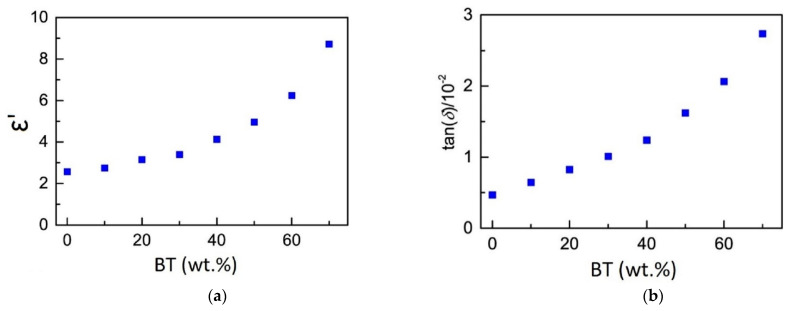
(**a**) Values of the permittivity ε′ of 3D printed parts for various loading fractions of BT in ABS; (**b**) Values of the loss tangent of 3D printed parts for various loading fractions of BT in ABS (available under Creative Commons license [58]).

A split-post dielectric resonator with a nominal measurement frequency of 15 GHz was used to determine the bulk permittivity of the 3D-printed parts. The authors used solid, disc-shaped test samples with a diameter of 35 mm and thickness of 0.5 mm for determining dielectric properties. The dielectric properties results of 3D-printed samples from single filament types with various loadings of BT powder are listed in Table 1 and plotted in Figure 3.

Based on test results, the authors found that the permittivity increased considerably as the BT fraction in the composite was increased, from 2.57 ± 0.02 for the unloaded polymer, to 8.72 ± 0.04 for the 70 wt.% polymer composite. The loss tangent also increased with increasing fraction of BT, from (0.469 ± 0.001) × 10^−2^ for the unloaded polymer, to (2.736 ± 0.012) × 10^−2^ for the 70 wt.% polymer composite.

**Figure 4:**
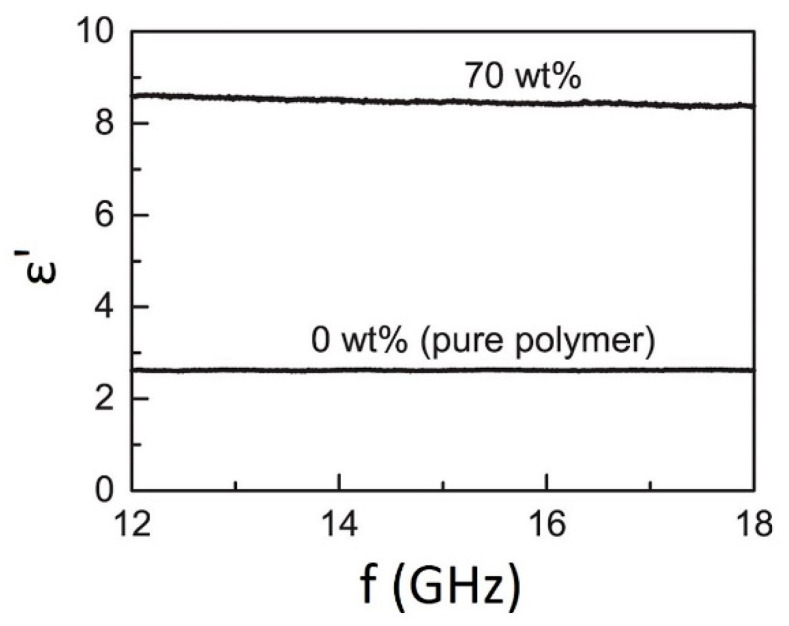
The permittivity ε′ as a function of frequency for ABS polymer only, and 70 wt.% BT/ABS polymer composite (available under Creative Commons license [58]).

For determining permittivity frequency dispersion authors investigated by carrying out measurements using a 7.9 mm × 15.8 mm rectangular waveguide with the Nichol-son-Ross-Weir extraction method. The 0 wt.% and 70 wt.% samples were chosen for this test. Test results data over the 12–18 GHz frequency range are presented in Figure 4. The authors reported that the 3D-printed samples exhibited relatively little dispersion over this frequency range. The permittivity was varied by less than 1.5%, even for the most highly loaded 70 wt.% samples.

In addition, the authors found that special attention should be paid to the control of FGP 3D-printing since the dielectric properties of the 3D-printed parts depend to a certain extent on the quality of the filament, and control over the printing process. In particular, permittivity will be reduced if any voids are trapped within the printer.

Multi-material FGP also finds application in resonator elements [72]. This area is promising in fabricating structures with electromagnetic properties of metamaterials and adjustable operating frequencies. Isakov et al. [57] demonstrated results where 3D-printed high dielectric rods arranged in a much lower permittivity matrix can be used as resonator elements, giving weaker interactions with applied fields but with potential for lower loss. Because AM can readily achieve these structures. Because AM can readily achieve these structures, the authors noted that the resonator approach based on dielectric arrays may offer some further flexibility in practical implementation. One example is producing the graded-index materials operating at microwave frequencies.

**Figure 5:**
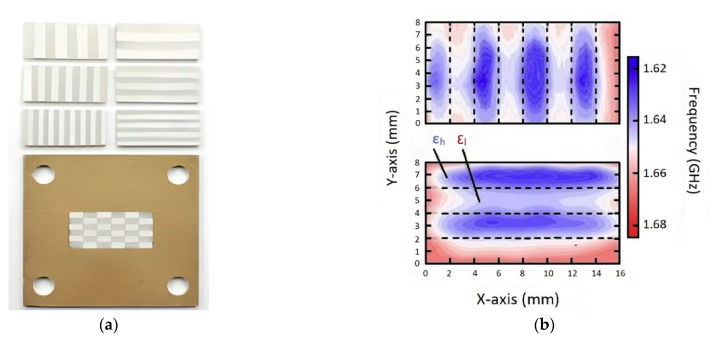
(**a**) Various printed coupons for dielectric characterization, comprising stripes or other arrangements of relatively low (ABS only) and high (dielectric ceramic/ABS composite) permittivity materials; (**b**) Experimental measurements of the directional resonant frequencies distribution of the split ring probe (Reprinted from Ref. [57], copyright (2016), with the kind permission of Elsevier).

To implement this approach, the authors printed gradient structure samples with relatively low and high permittivity regions. Two filament types were used simultaneously to print coupons comprising relatively low and high permittivity striped regions, such as ABS or PP for the low dielectric permittivity regions. In contrast, high dielectric permittivity regions used a mixed inorganic ceramic powder-polymer composite. Micro-particles of different perovskite oxides, such as BaTiO_3_, CaTiO_3_, and Ba_0.64_Sr_0.36_TiO_3_ (powder size < 3 μm) were added to the polymer matrices. As equipment, a dual-extrusion commercial FDM 3D printer (Makerbot Replicator 2) was used for the multi-material FGP. This 3D printer belongs to the material extrusion category. Figure 5 shows 3D-printed FGP samples which consist of vertically or horizontally arrays of alternating low (ABS only) and high (ABS + micro-particles) permittivity stripes. The chess-board lattice samples, with inter-leaved low and high permittivity materials, are also shown to demonstrate the capability of the AM of multi-material FGP. Figure 5b shows the result of the directional distribution resonant frequencies of the split ring probe in the eight-column (four filled, four unfilled) and four-row (two filled, two unfilled). The test results revealed that the surface probe had good sensitivity to the different printed regions of FGP.

The test results for dielectric properties for various materials are presented in Table 2. The maximum loading of ceramic powder that could be printed reproducibly was approximately 30 vol.%.

**Figure 6:**
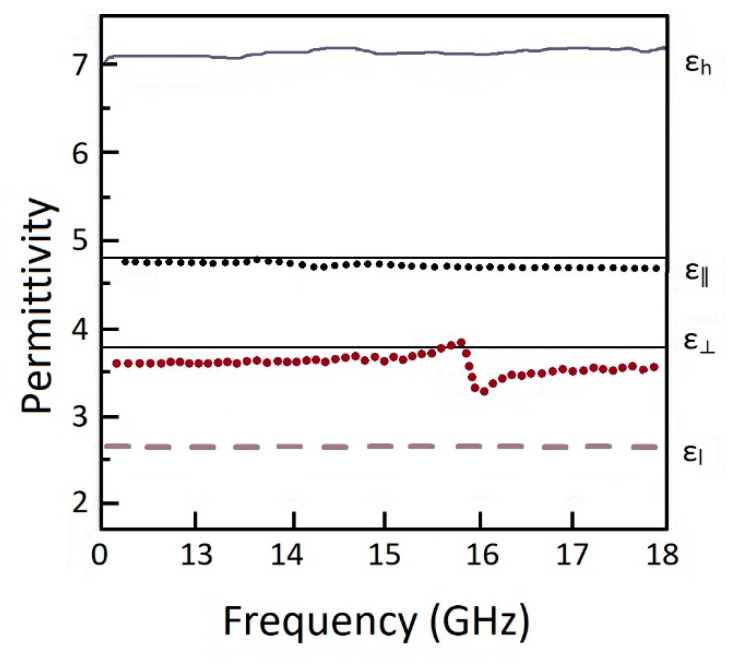
The test results of dielectric properties, where ε∥ is the impinging microwaves parallel to the long direction of the stripes; ε⊥ is the impinging microwaves parallel perpendicular to the long direction of the stripes; ε_h_ is the material with high permittivity (Table 2); ε_l_ is the material with low permittivity (Table 2) (Reprinted from Ref. [57], copyright (2016), with the kind permission of Elsevier).

The dependence results of the permittivity on frequency for the vertical and horizontal gradient regions are presented in the form of graphs in Figure 6. Data revealed that the ε⊥ plot has a perturbation in the effective permittivity at 15.9 GHz frequency. The authors noted a similar effect in all printed materials with alternate low and high permittivity number of layers in the case of two, three, and four-high permittivity stripes. It has been noticed that the perturbation amplitude became less pronounced as the width of the high permittivity stripe decreased.

Ultimately, the authors revealed the great potential for using FDM 3D printing in manufacturing novel electromagnetic devices, due to using multi-material FGP with near-zero or less than unity values of effective permittivity.

#### 2.1.2. Controlled Gradient Porosity of the FGP

In this section, we consider possible ways to produce an FGP with a controlled structure. The concept of “controlled structure” refers to the possibility of creating a periodic structure by designing cellular structures, or columnar elements, with a given direction and number. In the latter case, connectivity piezocomposites are a particular element in creating such an FGP.

**Figure 7:**
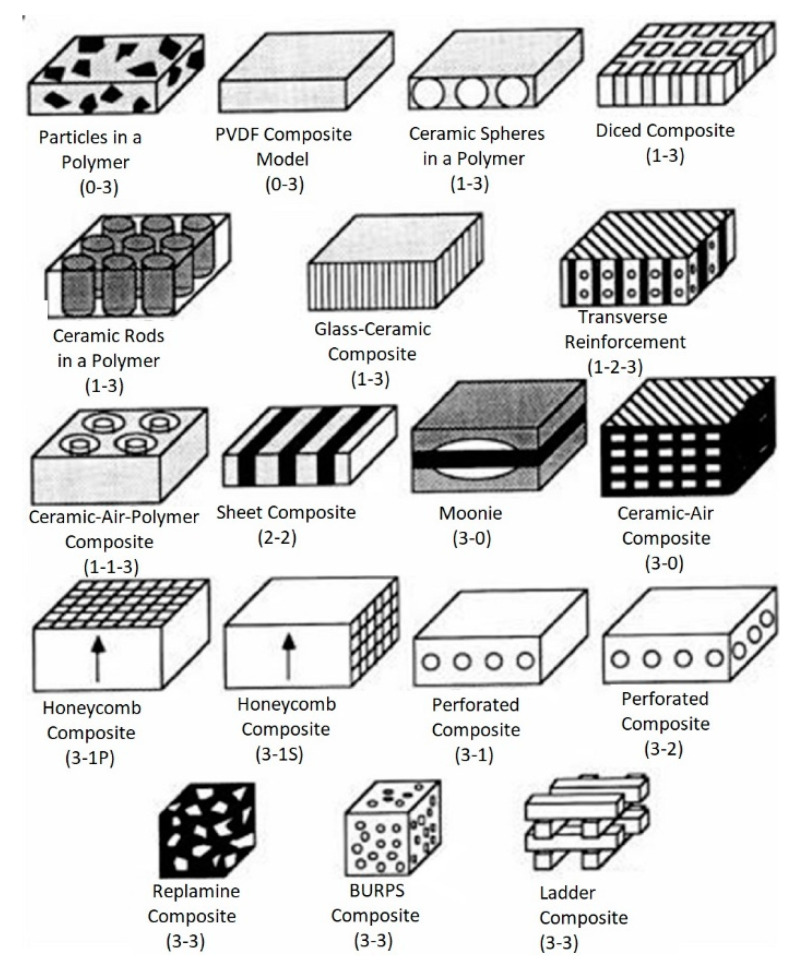
The examples of piezoelectric composites based on the diphasic connectivity concept (Reprinted from Ref. [48], copyright (2016), with the kind permission of Elsevier Ltd. and Techna Group S.r.l.).

The connectivity piezocomposites demonstrate superior properties to the single-phase materials [48]. Such connectivity piezocomposites are formed by combining at least two materials according to the desired product characteristics. There are 10 possible modes of connection in two-phase mixing: (0-0), (0-1), (0-2), (0-3), (1-1), (1-2), (2-2), (1-3), (2-3), and (3-3) (Figure 7). The first number indicates the connectivity of the active phase (piezoceramic), and the second number indicates the inactive phase (e.g., polymer). Some of these connection modes are particularly suitable for separating longitudinal and transverse piezoelectric effects, significantly enhancing the piezoelectric properties of the material. Through functional advantages such as a high dielectric constant, low dielectric loss, high energy storage density, and large breakdown strength the piezoelectric composite materials are important for emerging applications, such as wearable electronic devices, sensors, energy storage devices, and biomedical devices [73].

Various traditional processing techniques have been used to produce connectivity piezocomposites. The most widespread processing techniques are dice and fill, extrusion, injection molding, lost mold, tape lamination, dielectrophoresis, relic processing, laser or ultrasonic cutting, jet machining, reticulation, and co-extrusion. It is worth noting that the dice and fill, injection molding, and lost mold techniques are the most widely used methods for producing commercially available piezocomposites [74,75,76]. However, such technologies have one disadvantage related to mechanical stress that can lead to grain loss, strength degradation, and near-surface-area depolarization. Consequently, the performance of the piezoelectric element is significant reduction [73]. The formation of complex-shaped piezocomposites using traditional processing techniques is a difficult task, and it is impossible to impart a higher-order three-dimensional structure to brittle ceramics. AM makes it possible to obtain FGP with various surface geometries and different volumetric component concentrations.

The possibility of using 3D printing for FGP has been demonstrated in the manufacture of PZT/polymer composites, with various types of connectivity; therefore, the connectivity piezocomposite (2-2) can be made by the dicing technique or 3D printing. Using the traditional dicing technology poses some limitations related to fabricating a volume fraction gradient in the elevation direction of a composite (2-2). The so-called kerf width has to be increased from the center to the edges, while a cutting blade with a single thickness is used in the dicing machine [74].

**Figure 8:**
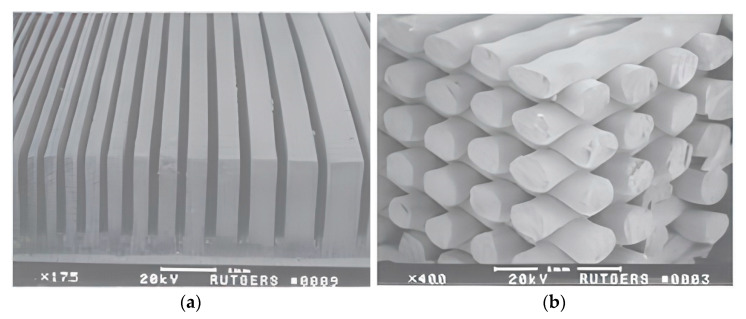
(**a**) 3D-printed connectivity piezocomposite (2-2); (**b**) 3D-printed connectivity piezocomposite (3-3) (Reprinted from Ref. [74], copyright (2007), with the kind permission of Springer Science + Business Media LLC).

In work [77], the authors demonstrated a solution to this difficulty through 3D printing, which produced ~60 vol.% ceramic in the center region of the composite to obtain a high acoustic pressure output at the center. The ceramic content was gradually decreased to approximately 20% at the edges of FGP composites. Due to the low dielectric constant of those regions, the edges would act as a good receiver of ultrasonic waves. The authors noted that a further decrease in ceramic content at the edges of FGP samples could further enhance the receiving sensitivity. Figure 8a shows an SEM image of the connectivity piezocomposite (2-2). After the sintering process, the gradient structures were embedded in epoxy.

The 3D printing process is also used for producing connectivity piezocomposite (3-3) based on the PZT/polymer with 3D honeycomb and ladder patterns [77]. Picture 8b shows an SEM image of a sintered ceramic ladder structure before epoxy infiltration. This structure was printed by the fused deposition of ceramics (FDC) process. The FDC process belongs to the material extrusion category, in which ceramic loaded thermoplastic filaments are fed into the liquefied. The ladder structure samples were built using a raster fill strategy with fixed inter-road spacing. The ceramic volume fraction in the ladder structure was approximately 70 percent. As noted by the authors the ceramic volume can be adjusted by varying the width and spacing between the ceramic roads. The ceramic roads are ~300 μm wide with ~800 μm center-to-center spacing. The FGP composites printed by FDC are highly uniform with excellent unit cell repeatability. The FDC process demonstrated superiority over traditional methods of composite production.

**Figure 9:**
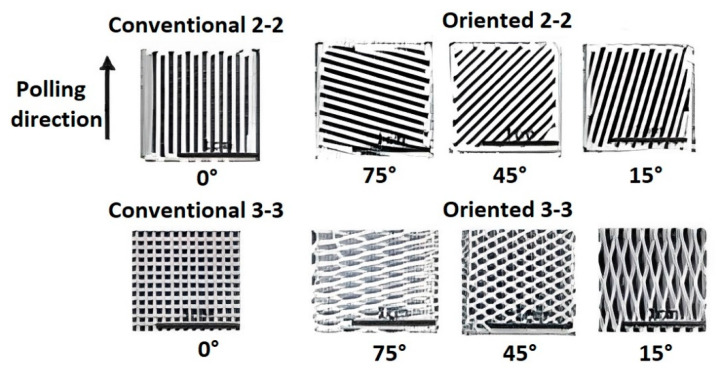
Oriented connectivity piezocomposites (2-2) and (3-3) printed by the FDM method (Reprinted from Ref. [74], copyright (2007), with the kind permission of Springer Science + Business Media LLC (New York, NY, USA, www.springer.com)).

Turcu et al. [78] studied PZT/epoxy ceramic-polymer composites (2-2) and (3-3) printed by the FDC process. A modified Stratasys FDM 3D printer was used for the printing of FGP. The green (2-2) and (3-3) piezocomposite structures were made with different orientation angles (θ) of the volume ceramic fraction concerning the poling direction (Figure 9). The orientation angle (θ) varied between 0° and 75° for both types of composites with 15° increments. The ceramic volume fraction was chosen to be 30 percent for both types of composites. Authors showed that when the ceramic walls deviate from the poling direction and the orientation angle is <15°, the (2-2) piezocomposites have a similar piezoelectric coefficient (d_33_) value to that of a classical composite (θ = 0). However, the d_33_ decreases abruptly from ~380 pC/N to ~200 pC/N when θ > 15°. For the (3-3) piezocomposites with θ < 45°, the deviation from poling direction does not affect the d_33_ values of composites. At θ > 45°, the d_33_ significantly decreases from ~450 pC/N to ~100 pC/N (θ = 75). Nan et al. [79] studied the effects of volume fraction and polarization orientation on the effective behavior for connectivity piezocomposite (0-3) and (1-3). The test results showed that the dielectric and piezoelectric properties of connectivity piezocomposite (1-3) decrease when the direction of polarization deviates from the orientation of the ceramic phase.

**Figure 10:**
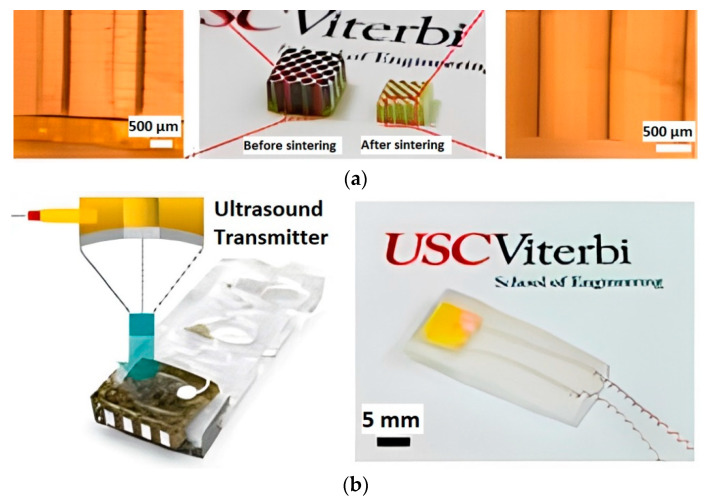
(**a**) The 3D-printed honeycomb structure of FGP before (left) and after (right) sintering; (**b**) Schematic and design of the ultrasonic device (left) and fabricated device (right) (available under Creative Commons license [80]).

Fabricating a more complex FGP composite with controlled gradient porosity finds application in ultrasonic devices. Zeng et al. [80] demonstrated 3D printing of piezoelectric composite based on BT with honeycomb structure by the Mask-Image-Projection-based Stereolithography (MIP-SL) process (Figure 10), which belongs to the vat photopolymerization category. The authors developed a tape-casting-integrated MIP-SL process based on the bottom-up projection approach, which can build ceramic green parts using slurries with high solid loadings. At the first stage the 3D-printed green models of FGP samples were debinded to remove the photocured resin. At the second stage the debinded FGP samples were sintered at 1350 °C for 4 h to create dense ceramic parts. The density of sintered part was 5.96 g/cm^3^. After sintering, each hole of the sintered sample with a honeycomb structure design was filled with epoxy to gain a composite material sample. The curing of filled epoxy was at 40 °C for 4 h. The top and bottom sides of the sample were then sputtered with Au/Cr electrodes for electrical connection. The sputtered sample was poled under a 20 kV/cm electrical field at 25 °C for 30 min. The honeycomb composite structure test results showed the piezoelectric constant equal to 60 pC/N. Finally, the sintered FGP sample was integrated into an ultrasonic device for ultrasound sensing. The test results demonstrated the output voltage amplitudes comparable ultrasound sensing performance of the 3D-printed sample where the maximum output voltage reached 180 mVpp.

**Figure 11:**
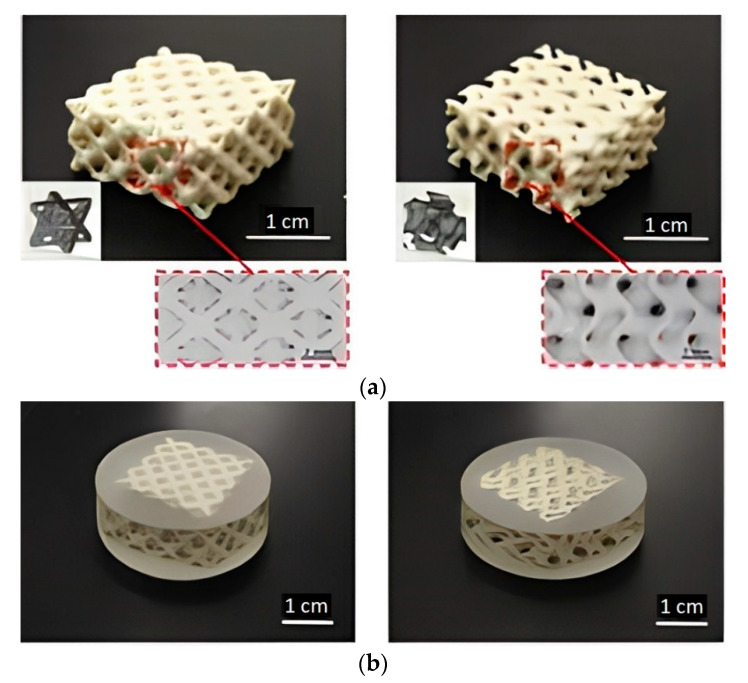
(**a**) Complex structures, octet-truss (left) and gyroid (right) of FGP fabricated by DLP method; (**b**) Piezoceramic/epoxy composites (Reprinted from Ref. [81], copyright (2021), with the kind permission of Elsevier Ltd.).

The lattice structure FGP produced by AM is promising in devices for receiving sound and ultrasound underwater (hydrophone). Liu et al. [81] printed two types of FGP complex structures: octet-truss, and gyroid (Figure 11) by the digital light processing (DLP) method. The FGP structures were printed using a Ceraform 100 (Longer 3D, Shenzhen, China) DLP 3D printer, with an ultraviolet (UV) source of λ = 405 nm and a light intensity of 8 mW/cm^2^. This 3D printer belongs to the vat photopolymerization category. The authors demonstrated this using these structures in functional devices, such as hydrophones.

BT powder with a d_50_ = 993 nm particle size was used as the initial piezoceramic material. The debinding process included two types. The first type included the carbonization process in a nitrogen atmosphere, heating up from room temperature to 600 °C at a rate of 1 °C/min, and finally, cooling down with the furnace. During the heating period, the temperature was kept at 200 °C, 300 °C, 400 °C, 450 °C, 500 °C, and 600 °C for 1 h separately. The second type included heating the carbonized body to 600 °C at 1 °C/min under an air atmosphere and kept at 450 and 600 °C for 2 h. Next, the ceramic FGP sample was sintered under an air atmosphere at 1330 °C for 2 h. After sintering, epoxy resin was filled inside sintered FGP sample to obtain a three-way connected piezoelectric composite material (Figure 11b). To achieve piezoelectric properties the FGP composite samples were polarized in a silicon oil bath at 60 °C for 15 min under a 2 kV/mm electric field. The maximum d_33_ was 45 pC/N. Finally, the authors tested a 3D-printed FGP composite using the proof-of-concept hydrophone that demonstrated receiving a signal provided by the signal generator to generate sound waves. The hydrophone detects the sound waves emitted by the speaker and converts them into electrical signals. When the input signal frequency changed within the range of 1–5 kHz, the frequency of the output signal also changed, and the magnitude was the same, which verifies the signal receiving function of the hydrophone.

**Figure 12:**
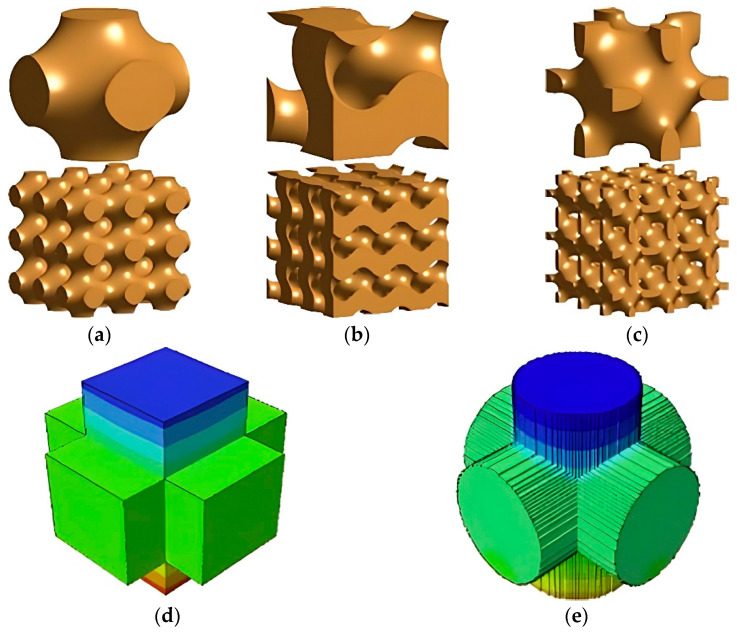
(**a**–**c**) TPMS structures (bottom) are composed of 3 × 3 × 3 unit cells such as Schwarz Primitive, Gyroid, and Neovius structures, respectively; (**d**) Electric potential distribution on the surface of the intersecting cuboids; (**e**) Electric potential distribution on the surface of the intersecting cylinders (Reprinted from Ref. [82], copyright (2020), with the kind permission of Elsevier Ltd. (Amsterdam, The Netherlands, www.elsevier.com).

Xu et al. [82] investigated the dependence of FGP output voltage on the ceramic volume fraction and structure of the ceramic component, together with the type of stimulus, using finite element analysis. The authors reported that when FGP composites are shaped into structures with a topology of triply periodic minimum surfaces (TPMSs) (Figure 12a–c), such as Schwarz Primitive surface, Gyroid surface, and Neovius surface, they exhibit much better piezoelectric performance than existing piezocomposites.

The authors used connectivity piezocomposite (3-3) as the initial sample for modeling. The interconnected cellular elements are improved by the piezoelectric characteristics of the piezocomposite. Also, PZT ceramic was used for modeling because of its strong piezoelectric properties, in response to external stimuli like pressure and vibrations. As a non-piezoelectric material, polydimethylsiloxane (PDMS) was used owing to its non-toxicity, elasticity, and durability. As a piezoelectric material, PZT ceramic was embedded in the PDMS matrix, allowing highly effective load transfer, large compression-induced piezoelectricity, good mechanical flexibility, and low degradation under loading cycles. The authors found that the output voltage of Schwartz P piezocomposite attains 802 V, 2007 V, and 3211 V under the compressive strain of 2%, 5%, and 8%, respectively. This is approximately 30% and 50% higher than cuboidal and cylinder piezocomposites (Figure 12d,e) at the same ceramic volume fraction (50%) (Table 3).

The high output voltage of the Schwarz piezocomposite is partially obtained from the large volume fraction (50%) of the piezoelectric component. The minimum ceramic volume fraction for connectivity piezocomposite (3-3) was 16%. In this case, the surface becomes discontinuous, and the output voltage is only 0.02 V. However, the Gyroid and Neovius surfaces are still continuous at such a low volume fraction. The authors compared output voltage for Gyroid, Neovius, and connectivity piezocomposites (1-3), (0-3), and (3-3). The output voltage values are presented in Table 4.

Based on the results in Table 4, the 0-3 piezocomposite output voltage is only 0.2 V under 8% compressive strain due to the highly discontinuous ceramic component. For the 1-3 piezocomposite, the connectivity of ceramic material was improved to some extent. As a result, its output voltage was improved to 26.87 V. The ceramic component became completely continuous in the 3-3 composite, and its output voltage is increased by ~3 times, approaching 84 V. The piezoelectric performance of these FGP composites illustrated that the ceramic material discontinuity plays a significant role in the piezoelectric effect. Without changes in connectivity, Gyroid piezocomposite can improve output voltage to 194.76 V, 486.91 V, and 779.05 V under the compressive strain of 2%, 5%, and 8%, respectively. Neovius piezocomposite had even better performance than Gyroid piezocomposite. The output voltage of Neovius attained 247.66 V, 877.12 V and 1210.33 V in the same compressive strains. On account of the output voltage being more than 17-fold and 6.000-fold larger than 3-3 piezocomposite and 0–3 piezocomposite under the 8% compressive strain, the authors have concluded that the shape of ceramic material also plays a significant role in enhancing the piezocomposite performance.

Also, the authors found that apart from volume fraction and shape, the piezoelectric performance also relies on the unit cell size. Under a compressive strain of 2%, the output voltage of Schwarz P piezocomposites with a unit cell width of 1 mm, 2 mm, 4 mm, 8 mm, and 10 mm is 802 V, 938 V, 1361 V, 1230 V, and 984 V, respectively. Numerical results indicated that maximum piezoelectric performance can be obtained at a specified size (e.g., the maximum output voltage of 1230 V of a piezocomposite with 4 mm unit cells). These results claim that piezoelectric performance will not enhance by constantly increasing the ceramic phase’s skeleton size.

#### 2.1.3. Disordered Gradient Porosity of the FGP

Porous graded ceramics significantly expand the functionality of materials due to their unique properties, namely, low density, high specific surface area, high impact strength, high-temperature resistance, good thermal insulation ability, and low dielectric constant, which are unattainable by their dense counterparts.

**Figure 13:**
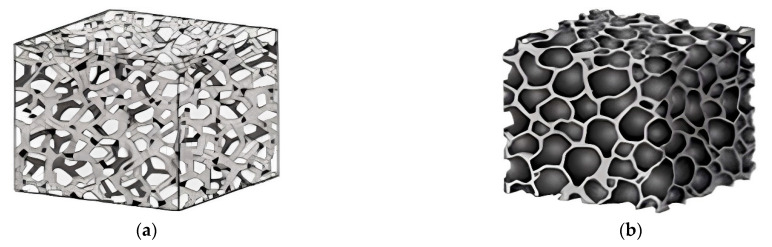
(**a**) Open pores of ceramic material; (**b**) Closed pores of ceramic material (Reprinted from Ref. [84], copyright (2007), with the kind permission of American Chemical Society).

Porous materials are classified into three types, depending on the pore diameter: macro-porous (d > 50 nm), meso-porous (50 nm > d > 2 nm), and micro-porous (d < 2 nm) [83]. Porous ceramics are characterized by pore type as open (or cellular) and closed pores (Figure 13), which largely determines the final functional properties of the material [84]. Open pores are distinguished into penetrating pores where fluid can penetrate, and non-penetrating pores are referred to as closed pores. Open pores are significant for separation-filtration, and closed pores are beneficial for lightweight and heat-insulating materials [85]. From the above, we can conclude that for a specific application, the right choice of a ceramic matrix with the required pore size and an open or closed pore structure is required, which will ultimately determine the final functional properties of the part.

Today, four mainstream approaches are widely investigated for manufacturing porous ceramics: partial sintering, replica template, sacrificial template, and direct foaming. The use of AM shows strong feasibility and unique advantages for producing porous ceramics, including FGP. Owing to the rapid layer-by-layer process according to a computer-aided design, precisely customized architectures with full control of the geometrical parameters, such as porosity, pore size, and pore interconnectivity, can be obtained by additive manufactured porous ceramics. Its worth noting that the pore size distribution of existing AM fabricated porous ceramics is on the microscale. The pore size distribution on the nanoscale will lead to a wide variety of energy and environment-related applications. Implementation of this process is achieved by combining AM with other pore forming strategies, such as partial sintering and sacrificial templates [86].

Disordered graded porous ceramics find wide applications in various fields of technology, such as: energy storage and conversion media applications (components in fuel cells and batteries, solar energy absorption and conversion); energy harvesting and sensing applications (electronic sensing applications. piezoelectric energy harvesting, pyroelectric energy harvesting); catalyst support applications (micro-reformer for hydrogenation process, photocatalyst for environmental remediation); insulation applications (sound-proof applications, electro-magnetic wave shielding); and filtration applications (water treatment, hot gas filters, diesel particulate filters).

Disordered porous FGP finds application in the fields of energy harvesting and insulation applications. High-permittivity ceramic matrix with a low permittivity pore added has a broad electric field distribution in the ceramic component and reduced electric field experienced by the ceramic due to an electric field concentration in the lower permittivity pore region [87]. Roscow et al. [88] fabricated macro-porous BT ceramics with a range of porosities, using the burned out polymer spheres (BURPS) process. Polyethylene glycol is used as a volatile pore-forming agent. The porosity of the material was 60%. Pores were spherical with diameters ranging between 100 and 400 μm. The energy harvesting figure of merit (FOM) significantly increased with the inclusion of porosity into the BT. The maximum value was 2.85 pm^2^/N at ~40% relative density compared with ~1.0 pm^2^/N for the dense material. This significant improvement in the energy harvesting FOM resulted from a large reduction in permittivity at this porosity level, and a relatively small reduction in d_33_. According to the research results, it was confirmed that for a given applied stress, the energy generated within a material with an appropriate pore volume fraction can be significantly larger than its dense counterpart. The challenge is to design and fabricate the appropriate pore configurations.

Reducing the relative density via the introduction of porosity reduces the strain coefficient, although above ~40%, the response remains relatively high at ~75% of the maximum d_33_ values achieved for dense BT. Reducing the relative density via the introduction of porosity reduces the strain coefficient above ~40%, and the response remains relatively high at ~75% of the maximum d_33_ values for dense BT. In high porosity samples (<40% relative density) the d33 decreased due to incomplete poling of the BT phase. On the one hand, this effect is caused by lower breakdown strength that occurs in highly porous dielectric ceramics and on the other hand complex electric field distribution due to a mixture of low and high permittivity phases [88,89]. Moreover, pores probably increase stress concentrations that lead to material depolarization and limit the grain size in their immediate vicinity. This is thought to be a result of inhibiting domain motion, both of which may reduce the achievable level of polarization.

**Figure 14:**
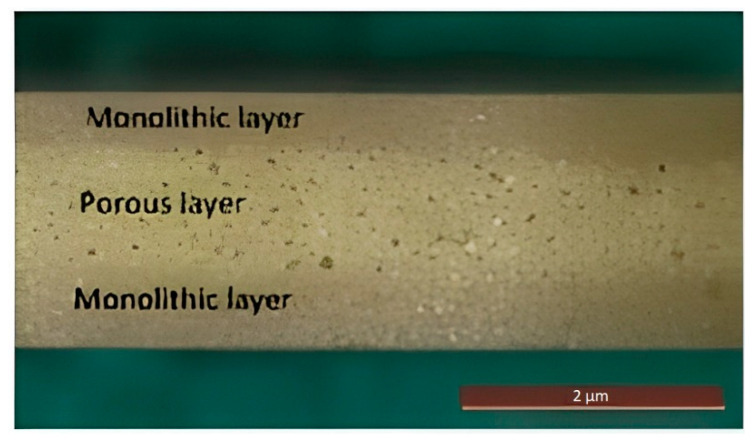
Porous multilayer BT material (Reprinted from Ref. [90], copyright (2017), with the kind permission of Acta Materialia Inc. Published by Elsevier Ltd.).

Also, a disordered porous FGP finds application in piezoelectric energy harvesting, where the choice of material with low stiffness allows a more efficient transfer of mechanical energy to the material. Roscow et al. [90] fabricated layered BT ceramic (Figure 14), whereby dense outer layers surround a highly porous sandwich layer.

According to the research results, the FOM value has reached 3.74 pm^2^/N, which is a factor of 2.65 higher than the value for dense BT (FOM = 1.40 pm^2^/N). In addition, porous BT was fabricated with a porosity of 45%, where a maximum voltage of 234 mV was achieved compared to 96 mV for dense BT, representing a 2.4 times improvement. Such improvements in material properties show the high potential of porous FGP for high performance energy harvesting.

**Figure 15:**
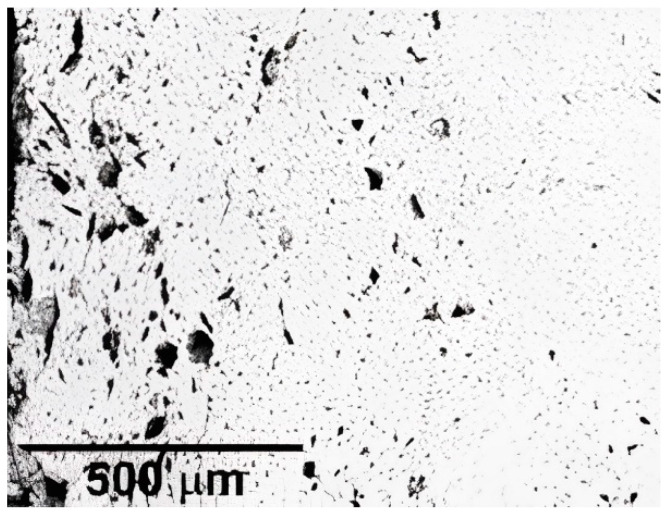
Layered FGP with graded porosity (Reprinted from Ref. [91], copyright (2005), with the kind permission of Elsevier Ltd.).

Piazza et al. [91] demonstrated the results of producing FGP with increasing porosity content to a maximum of 40 vol.%. Graded porosity was obtained by adding lamellar graphite as a pore forming agent (PFA) with different content (0, 5, 10, 20, and 40 vol.%) to piezoceramic powder based on the composition Pb(Zr_0.52_Ti_0.48_)_0.976_Nb_0.024_O_3_. The authors used graded porosity disks (25 mm diameter) produced by die pressing five layers at increasing graphite content. The thicknesses of the thin and thick disk samples were 2.5 mm and 1.2 mm respectively. The samples were sintered at 1150 °C for 2 h. Homogeneous and FGP samples from every batch of powder were ground to remove surface layers. Next, the samples were screen-printed with silver electrodes, then fired at 700 °C, and finally poled into silicon oil at 120 °C, under a direct current field of 2 kV/mm for 40 min. The data of the functional properties at different PFA content are presented in Table 5. The FGP microstructure (Figure 15) is the sum of the effects of the different graphite contents with the low sintering temperature. In addition, during the sintering process, the five layers’ structure is cancelled, developing a continuously grading porosity without growing cracks or delamination at the interfaces between the different layers.

Based on data from Table 5 the values of the piezoelectric constants showed that the functional properties decay at increasing porosity content. Moreover, the piezoelectric properties in the thickness direction (d_33_, k_t_) decrease less than the properties in the planar direction (d_31_).

A similar dependence of the piezoelectric properties on the material porosity was demonstrated in work [92], where using lead zirconate titanate-lead cobalt niobate (PZT-PCN) powder as the main material and the polyvinyl chloride (PVC), stearic acid (SA) and polymethyl methacrylate (PMMA) as PFA to create porosity in the samples. The FGP samples were fabricated by BURPS with different content of three PFA of PMMA (1%, 3%, and 5%), PVC, and SA (5%, 10%, and 15%). Then the prepared compacts were sintered for 2 h at 1200 °C using a covered alumina crucible pitted with PZT-PCN powder to reduce the volatilization of PbO. The surface layers of the sintered samples were removed by a grinding process. Next, the samples were evaporated in a vacuum using silver electrodes. Finally, the samples were poled via a direct current field of 2–3 kV/mm for 10 min within a silicone oil bath at a temperature of 120 °C. The measured dielectric and piezoelectric properties are presented in Table 6 for both dense and porous piezoelectric PZT-PCN ceramics.

**Figure 16:**
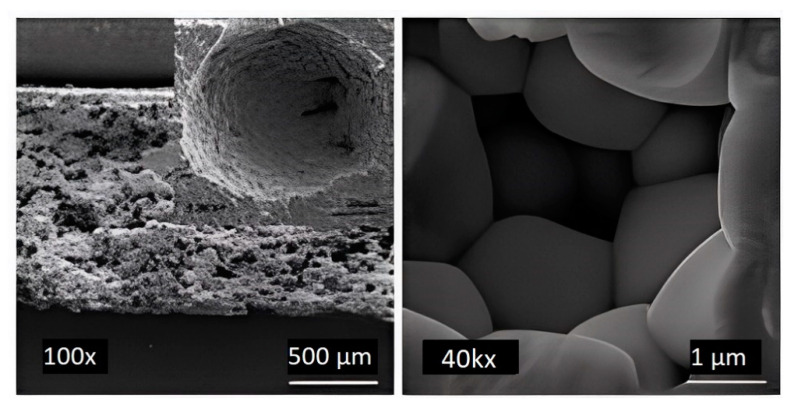
SEM images of produced porous PZT-PCN ceramic synthesized by 5 wt.% of PMMA as PFA (Reprinted from Ref. [92], copyright (2019), with the kind permission of Elsevier Ltd.).

Thus, the authors concluded that the d_33_ and ε_r_ decrease due to enhancing the porosity of the PZT-PCN ceramics. Also, the authors managed to obtain a value of 2732 × 10^−15^ m^2^/N for the hydrostatic FOM pertaining to porous PZT-PCN ceramics with spherical pores prepared using 5 wt.% PMMA (Figure 16), which demonstrates that porous PZT-PCN ceramic is a promising material for biomedical diagnostic devices.

## 3. Binder Jetting 3D Printing of FGP

In 1989 the BJ 3D Printing was first designed at the Massachusetts Institute of Technology (MIT). The BJ process includes the main steps such as spraying the organic binder solution through printheads onto selected regions of a powder bed surface; forming the solid layers by the solidification (i.e., glueing) of the permeating liquid binder; supplying a new powder layer on the previous layer to repeat the building process until the part is formed. Previously, an exclusive license for ceramic BJ was held by the Soligen company. However, machines for the ceramic BJ are more popularly commercialized by companies such as ExOne and 3D Systems [93].

**Figure 17:**
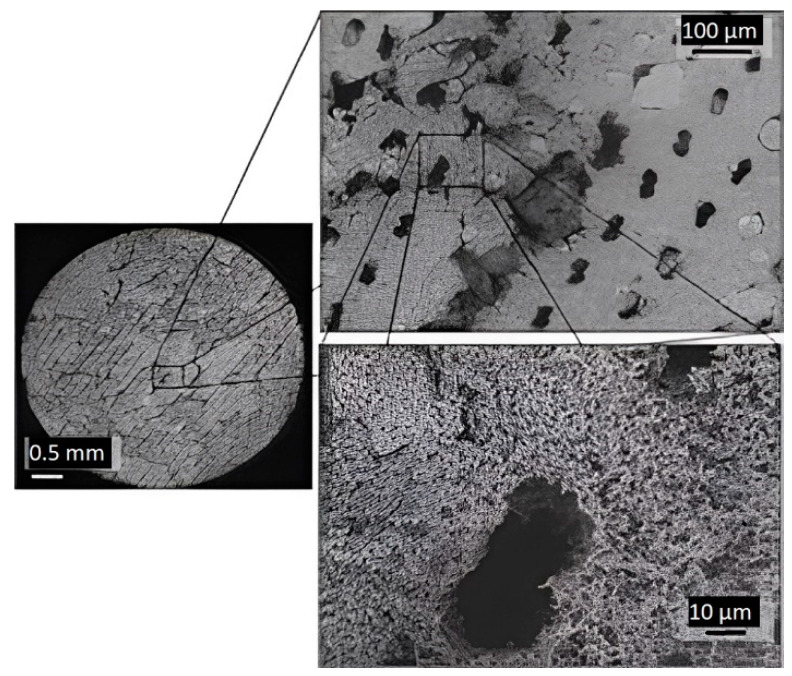
Sequential set of images showing the presence of the voids left from the binder that has been burned out after heating the sample at 1200 °C for 4 h (Reprinted from Ref. [99], copyright (2015), with the kind permission of Elsevier Ltd. and Techna Group S.r.l.).

The use of BJ is also possible in the 3D printing of FGP parts [94,95,96,97,98]. Gaytan et al. [99] presented the results of the 3D printing of BT-based piezoceramic material by the BJ technology. Spherical BT powder (0.85–1.45 μm) was used for fabrication samples. The FGP samples were printed by the BJ technology using a commercial ExOne M-Lab system. The ExOne M-Lab printer consists of a two-bed system; one bed is for the base powder material and the other for part fabrication. The build volume of the ExOne M-Lab printer is 40 × 60 × 35 mm. Fabrication of BT was performed using a layer thickness of 30 μm since smaller layers would not provide a uniform powder distribution, due to agglomeration. For binder curing, the building envelope is placed in the oven at 195 °C for 2 h. The debinding process was carried out at 600 °C for 20 min. Sintering of FGP was implemented by heating the samples at 1260, 1330, and 1400 °C for four hours with a ramp-up rate of 10 °C/min. The temperature was decreased at intervals of 100 °C with dwell times of one hour after reaching the maximum value. The results showed a maximum theoretical density of 65% (3.93 g/cm^3^) obtained by sintering at 1400 °C for four hours. The shrinkage for samples was in the range of 20–30%. The relatively low density and high shrinkage were mainly due to the droplet nature of the binder material deposition. Figure 17 shows SEM images of the material structure after sintering, showing voids in the sample formed by droplets of binder that burned out after heating.

**Figure 18:**
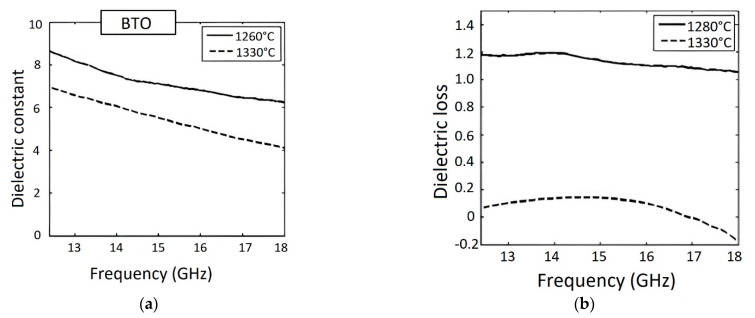
(**a**) Dielectric constant and (**b**) Dielectric loss for BT sintered at 1260 and 1330 °C for a frequency range of 12.4–18 GHz (Reprinted from Ref. [99], copyright (2015), with the kind permission of Elsevier Ltd. and Techna Group S.r.l.).

**Figure 19:**
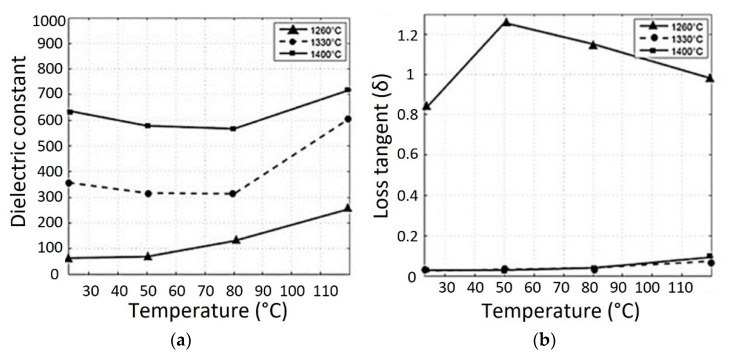
(**a**) Dielectric constant and (**b**) Dielectric loss for BT sintered at 1260, 1330, and 1400 °C at 1 kHz frequency (Reprinted from Ref. [99], copyright (2015), with the kind permission of Elsevier Ltd. and Techna Group S.r.l.).

The authors consider the printed piezoceramics is an ideal candidate for sensor applications, so the piezoelectric properties were measured and amounted to d_33_ = 74.1 pC/N for samples sintered at 1400 °C and only 13.23 pC/N for samples sintered at 1260 °C. Additionally, the dielectric constant measurements for printed BT ranged from 8.62 to 6.23 for a frequency range of 12.4–18 GHz, respectively, for the sample sintered at 1260 °C. For the same frequency range, dielectric constant values of 6.95–4.09 were obtained for samples sintered at 1330 °C. The dielectric loss was also lower for samples sintered at 1330 °C. Figure 18 shows the dielectric constant and the loss obtained for BT sintered at 1260 and 1330 °C for a frequency range of 12.4–18 GHz. Figure 19 shows the dielectric constant and the dielectric loss for BT sintered at 1260, 1330, and 1400 °C for a frequency of 1 kHz.

The graphs show that the dielectric constant increases with an increase in the sintering temperature of the piezoceramic material. However, Vijatovic et al. [100] demonstrated results where the dielectric permittivity for BT decreases with increasing grain size. The authors noted that the grain size and porosity could be improved by preparing samples using the BJ method. The dielectric constant can be increased for ultracapacitors or three-dimensional antenna structures. However, the dielectric permittivity values are frequency dependent, independent of the sintering temperature and part density.

**Figure 20:**
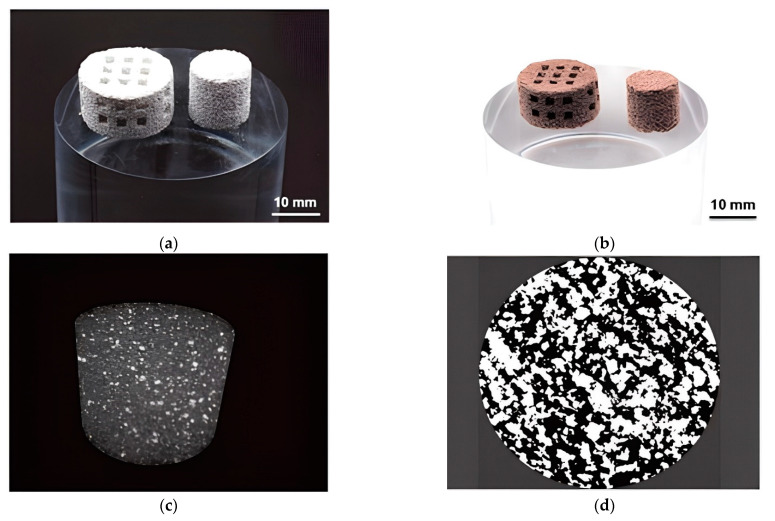
(**a**) FGP samples (scaffolds) with controlled gradient porosity produced by BJ after 3D printing; (**b**) After sintering; (**c**) Three-dimensional maximum intensity projection of FGP samples (scaffolds); (**d**) The binarised cross-sectional microCT images reveal the number of pores (black) (available under Creative Commons license [101]).

The production of FGP with controlled gradient porosity by the BJ technology was presented in work [101]. Authors printed ceramic scaffolds with interconnected porosity for bone tissue engineering from a composition containing 68 wt.% BT, 18 wt.% hydroxyapatite (HA), and 14 wt.% poly(ethyl methacrylate) (PEMA). The samples were printed from composite powder by a commercial 3D printer, VX500 (Voxeljet Technology GmbH, Augsburg, Germany). As a binder was used SOLUPOR partially dissolves the polymeric phase to glue the ceramic particles together. The SOLUPOR binder is a solvent mixture consisting of hexane-1-ol, 2-ethylhexyl acetate, and hexyl acetate. The FGP samples were dried for 24 h at 40 °C. The polymeric phase was debinded via pyrolysis from the scaffolds by heat-treating in a debinding furnace at 300 °C for 1 h and at 500 °C for 2 h. After debinding, the FGP green models were sintered at 600 °C, 1000 °C, and 1320 °C in the atmosphere. After sintering at 1320 °C, the scaffolds show a strong shrinkage of about 28.4 ± 0.93% in volume but maintain their geometrical shape (Figure 20).

The printed FGP samples were polarised, starting from 0.667 kV/mm to 1.25 kV/mm. The maximum piezoelectric coefficient d_33_ was 3.08 pC/N after polarization at 1.25 kV/mm field strength. This material limits the 3D-printed samples for applications such as sensors or energy harvesting, but is suitable for using the indirect piezoelectric effect to enhance bone growth. The authors observed cellular adhesion, suggesting that the 3D-printing process of BT/HA presents microstructural features and chemical compatibility suitable for osteoblast-like cell attachment and growth. The results demonstrated that the AM of lead-free BT-based FGP by BJ represents a promising approach to yield scaffolds of designed porosity equipped with piezoelectric properties for enhanced bone regeneration.

**Figure 21:**
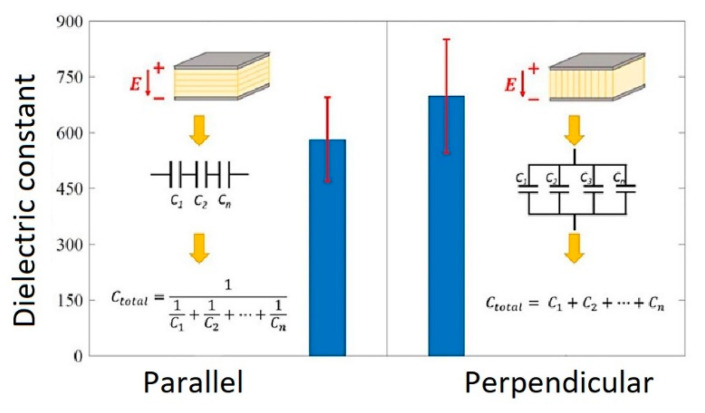
Comparison of average dielectric constant and standard deviation at 1 kHz (Reprinted from Ref. [102], copyright (2019), with the kind permission of IOP Publishing Ltd.).

**Figure 22:**
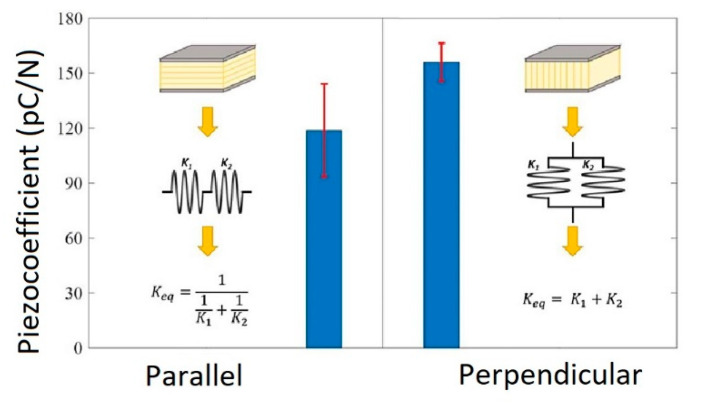
Comparison of piezoelectric coefficients and standard deviation between sample orientations (Reprinted from Ref. [102], copyright (2019), with the kind permission of IOP Publishing Ltd.).

Chavez et al. [102] fabricated and characterized 3D printing induced orthotropic functional piezoceramic, produced by BJ technology. The authors demonstrated the results of the dielectric and piezoelectric properties of these printed piezoceramics as a function of the printing orientation. As the raw material, BT spherical powder with a maximum particle size of 45 μm was used to print samples. ExOne M-Lab R1 3D printer was used to fabricate the 3D objects. Additionally, the BS004 solvent binder and CL001 cleaner (The ExOne Company, North Huntingdon, PA, USA) were used to print the functional ceramic components. After the 3D printing step, the samples’ powder bed was cured at 200 °C for 2 h. Further, the cured samples were debinded at 650 °C for 1 h and sintered at 1250 °C for 6 h to densify the printed ceramics. After cutting samples in parallel and perpendicular planes to the printing direction, the surfaces were polished and then electroded using conductive silver paint. Finally, the samples were poled under a DC electric field of 0.33 kV mm^−1^ at a temperature of 60 °C in a silicone oil bath for 2 h. The dielectric and piezoelectric properties depending on the 3D printing direction are presented in Figure 21 and Figure 22, respectively.

The test results demonstrated that the average dielectric properties of the perpendicularly tested samples were 20% higher than those parallel to the printing orientation. The average dielectric constant of perpendicularly and parallel samples achieved the values of 698 and 581.6, respectively. This dielectric property dependence on the printing direction is explained by the morphology of the sample and printing layers, and the equivalent capacitor circuit formed in the different samples, as shown in Figure 21. In the case of the samples tested in the parallel direction, air pockets are formed between the high dielectric material layers, creating a set of capacitors arranged in a series circuit. Due to the low dielectric constant of air, this type of arrangement will inhibit the dielectric capacitance of the system, lowering the overall dielectric property in the system. In contrast, the perpendicularly tested samples created a set of capacitors arranged in a parallel circuit. Therefore, the air presence in between the layers did not negatively impact the dielectric performance.

**Figure 23:**
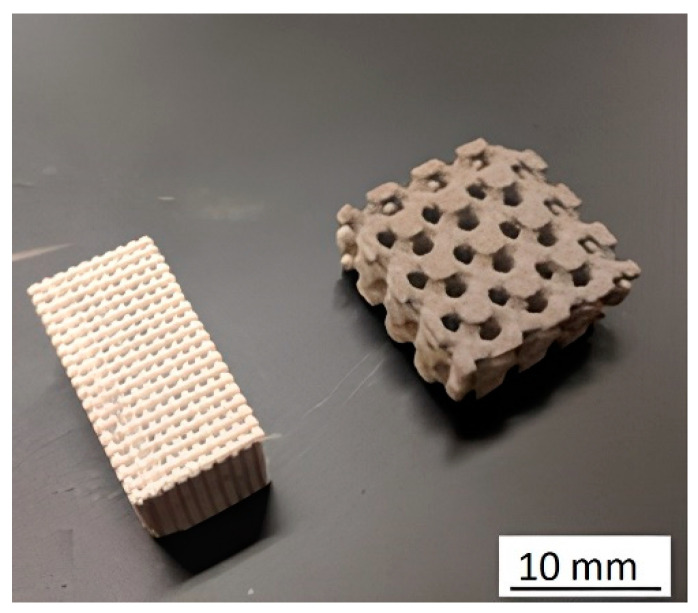
3D-printed FGP samples with controlled gradient porosity by the BJ technology (Reprinted from Ref. [102], copyright (2019), with the kind permission of IOP Publishing Ltd.).

Similarly, a dependence on the fabrication orientation for the piezoelectric properties was observed where the average piezoelectric charge coefficient for the parallel samples was 113 pC/N. For the perpendicular samples, the piezoelectric charge coefficient was 152.7 pC/N. This difference in piezoelectric performance improved 35.1% over the parallel tested group. The printing direction influence on the piezoelectric properties is explained that for parallel electroding, mechanical loading was absorbed by the defects between printing layers, thus leaving the active piezoelectric ceramic not excited. To demonstrate the applicability of the BJ technology, the FGP samples with controlled gradient porosity were made from BT powder, shown in Figure 23.

**Figure 24:**
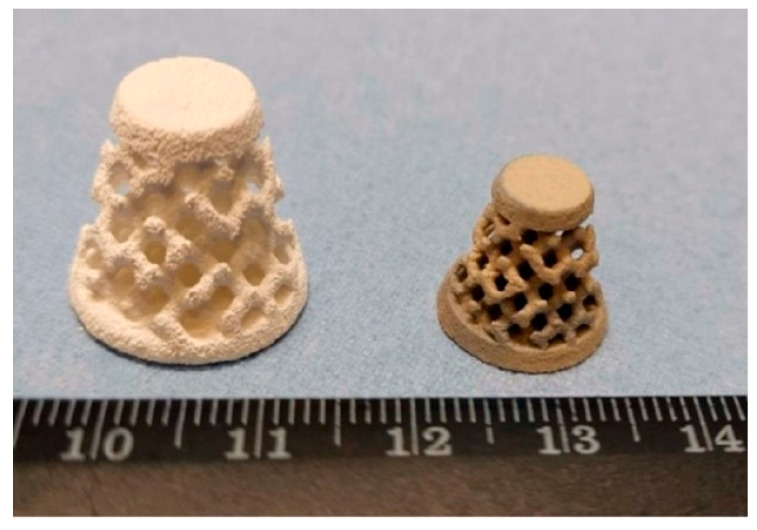
Image of FGP samples with controlled gradient porosity printing by BJ before (left) and after sintering (right) (available under Creative Commons license [94]).

Sufiiarov et al. [94] demonstrated the results of 3D printing samples from BT by using the BJ technology. The FGP samples were printed on a commercial 3D printer, ExOne Innovent system (The ExOne Company, North Huntingdon, PA, USA), using the original ExOne BS004 solvent binder and CL001 cleaner. The build volume of the ExOne Innovent printer is 160 × 65 × 65 mm. Two powders with different particle size distributions (PSD) (micron powder with multimodal PSD and submicron powder with unimodal PSD) were used as raw materials. The authors determined that samples from the unimodal powder are more sensitive to increasing grain size during sintering. The dielectric and piezoelectric properties of samples also demonstrated that samples from unimodal BT powder have higher values. The results of the functional piezoelectric properties obtained by BJ with multimodal PSD are d_33_ = 118 pC/N, έ = 750, and with unimodal PSD—d_33_ = 183 pC/N, έ = 811. To demonstrate the applicability of the BJ technology, the FGP samples with controlled gradient porosity were made from submicron powder with unimodal PSD (Figure 24).

The authors claim that the presented results demonstrate that using for printing a unimodal BT powder allows for achieving higher piezoelectric properties. The BJ process allows the manufacturing of complex geometry objects, which has potential in the production of ultrasonic devices used in medicine, aviation, the marine industry, sensors for monitoring welded joints, and pressure sensors in pipelines, etc.

## 4. Conclusions and Future Prospects

In this review, we focused on analyzing possible ways to create and control functional characteristics for graded piezoceramic materials, with various types of FGMs manufactured by AM. We proposed an FGP classification by gradient types, such as gradient chemical composition or multi-material 3D printing, and gradient porosity—controlled and disordered porosity. The review showed that FDM, DLP, SLA, and BJ are the most promising methods for 3D printing FGP. Additionally, we reviewed the current state of the BJ process 3D printing of FGP.

Based on this analysis, we found that the final functional material properties are determined mainly by the gradient type. In addition to the general classification presented above, we introduced an additional criterion according to the FGMs type. Namely, the “porosity” gradient type is further divided into two criteria: “controlled porosity” and “disordered porosity”. The research leads to the following conclusions:

(1) The gradient chemical composition of the FGP (multi-material 3D printing) is the most studied research area. Various options for 3D printing FGP elements of a simple shape are proposed, and the effect of a gradient chemical composition on the piezoelectric and dielectric properties is studied. For instance, 20% vol. BT produce dramatic material property variations in pure PZT (a 140% increase in permittivity (4022 for pure PZT to a plateau of approximately 9642) coupled with a 96% reduction in the piezoelectric effect (the d_31_ coefficient dropping from 283 pm/V to 6 pm/V)). It was established that when the resulting piezoelectric strain gradient is coupled with the variation in permittivity, the two gradients work synergistically to increase the deformations and electric efficiency of the FGP actuator [71]. Regarding ceramic/polymer FGP, the permittivity increases considerably with increasing BT fraction in the composite, from 2.57 ± 0.02 for the unloaded polymer to 8.72 ± 0.04 for the 70 wt.% polymer composite. The loss tangent also increases with increasing BT fraction, from (0.469 ± 0.001) × 10^−2^ for the unloaded polymer to (2.736 ± 0.012) × 10^−2^ for the 70 wt.% polymer composite [58]. However, it should be noted that creating a complex FGP shape with a gradient chemical composition requires high-tech equipment, namely, 3D printers with the ability to implement multi-material printing.
(2) FGP with a controlled gradient porosity (a periodic cellular or columnar structure) is a piezocomposite, with various types of connectivity. Such composites are formed by combining at least two materials according to the final product’s required characteristics; they also have more improved piezoelectric and dielectric properties than single-phase materials. The common connectivity piezocomposites are types (1-3), (2-2), and (3-3). For a connectivity piezocomposite (3-3), it is possible to use Schwarz, Gyroid, and Neovius surfaces as a periodic cellular structure. For instance, under 8% compressive strain, the output voltages of the traditional 0-3, 1-3, and 3-3 piezo composites are 0.2 V, 26.87 V, and 84 V, respectively. Gyroid and Neovius piezo composites can improve output voltages to 779.05 V and 1210.33 V, respectively, under the same compressive strain [82]. It should be noted that the creation of FGP with a controlled gradient structure is not limited to existing 3D equipment and can be implemented on commercially available 3D printers.
(3) The creation of FGP with disordered gradient porosity by AM is the least studied gradient type. However, such materials have a unique set of properties: low density, high specific surface area, high impact strength, high-temperature resistance, good thermal insulation ability, and low dielectric constant, which are unattainable by their dense counterparts. For instance, introducing porosity into the BT significantly increase the energy harvesting FOM, with a maximum of 2.85 pm^2^/N obtained at ~40% relative density compared with ~1.0 pm^2^/N for the dense material [88]. FGP with disordered gradient porosity finds application in energy. However, it should be noted that the production of 3D-printed FGP with disordered gradient porosity is possible by combining AM and traditional pore formation methods, such as partial sintering and the addition of a pore forming agent.
(4) Regarding piezoceramic materials for 3D-printed FGP, today the widely used materials are based on barium titanate, sodium-potassium niobate, and lead zirconate titanate. The most promising methods of 3D printing FGP are the FDM process (material extrusion category), DLP, stereolithography (SLA) (vat photopolymerization category), and BJ process (binder jetting category).

Despite AM advantages, 3D printing of FGP faces challenges in the area of thermal post-processing. The thermal post-processing needs to be done to remove the binder materials and maintain dimensional accuracies. For vat photopolymerization based technology, debinding is a key step as this process directly affects the final result of the subsequent sintering. We expect a good share of future research to explore these limitations. Regarding binder jetting-based technology, FGP samples have the lowest piezoelectric coefficient values and have a low density. We expect researchers to focus more on methods and ways of improving the density, that in turn improves FGP performance.

Based on the analysis of possible ways to produce an FGP by AM and the possibility of controlling the functional characteristics of such materials, it should be noted that this research area is relevant and promising for the use of FGP in the field of energy harvesting devices, sensors, use as insulators, which is highly relevant for the aviation industry in the manufacturing of aircraft radio equipment; in the maritime industry in the manufacture of transceiver modules for hydroacoustic antennas; and in the nuclear industry in the manufacture of sensors for steam-water path pressure monitoring. However, the literature analysis showed that today the creation of FGP by AM is a poorly-studied but promising area of research, due to the rapid development of the additive technologies market and their unique features in the shaping of parts.

## Figures and Tables

**Table 1:** Dielectric properties of 3D-printed polymer composite with various loadings of BT in ABS (available under Creative Commons license [58]).

BT (wt.%)	BT (vol.%)	ε′	tan (δ)/10^−2^	f (GHz)
0	0	2.57 ± 0.02	0.469 ± 0.001	14.83
10	1.9	2.75 ± 0.02	0.643 ± 0.003	14.82
20	4.1	3.15 ± 0.01	0.827 ± 0.003	14.74
30	6.8	3.40 ± 0.01	1.012 ± 0.004	14.74
40	10	4.13 ± 0.01	1.240 ± 0.005	14.63
50	15	4.95 ± 0.01	1.622 ± 0.001	14.55
60	20	6.24 ± 0.03	2.064 ± 0.005	14.42
70	29	8.72 ± 0.04	2.736 ± 0.012	14.13

**Table 2:** Printed materials and their estimated and measured dielectric permittivity and losses at 15 GHz (Reprinted from Ref. [57], copyright (2016), with the kind permission of Elsevier).

Material	Solid Loading (vol.%)	ε_r_	tan δ
ABS + BaTiO_3_	0.27	7.0	3.42 × 10^−2^
ABS + Ba_0.64_Sr_0.36_TiO_3_	0.30	6.7	3.68 × 10^−2^
PP + CaTiO_3_	0.27	5.0	5.10 × 10^−3^
ABS	-	2.65	4.80 × 10^−3^
PP	-	2.25	2.65 × 10^−4^

**Table 3:** Output voltage of piezocomposites with 50% PZT ceramic under the compressive strain (Reprinted from Ref. [82], copyright (2020), with the kind permission of Elsevier Ltd.).

Piezocomposite	ε_c_ = 2%	ε_c_ = 5%	ε_c_ = 8%
Cuboidal	535	1338	2142
Cylinder	605	1514	2422
Schwarz P	802	2007	3211

**Table 4:** Output voltage of piezocomposites with 16% ceramic volume fraction (Reprinted from Ref. [82], copyright (2020), with the kind permission of Elsevier Ltd.).

Piezocomposite	Compressive Strain		
	ε_c_ = 2%	ε_c_ = 5%	ε_c_ = 8%
Neovius	247.66	877.12	1210.33
Gyroid	194.76	486.91	779.05
piezocomposite (3-3)	21.00	52.00	84.00
piezocomposite (1-3)	6.72	16.79	26.87
piezocomposite (0-3)	0.05	0.13	0.2

**Table 5:** Physical and electrical properties of the samples at different porosity (Reprinted from Ref. [91], copyright (2005), with the kind permission of Elsevier Ltd.).

Graphite (vol.%)	Density (g/cm^3^)	Porosity (vol.%)	k_31_	k_t_	d_31_, (×10^−12^_m/V)_	d_33_, (×10^−12^_m/V)_	ε_33_^T^
0	7.58	5.4	−0.36	0.51	−179	396	1649
5	7.30	8.9	−0.31	0.51	−144	371	1369
10	6.98	12.8	−0.26	0.42	−120	351	1153
20	6.39	20.2	−0.13	0.47	−51	312	730
40	4.95	38.2	−0.04	0.17	−15	202	404

**Table 6:** The measured density, porosity, piezoelectric and dielectric properties of porous PZT-PCN ceramics (Reprinted from Ref. [92], copyright (2019), with the kind permission of Elsevier Ltd.).

PFA	PFA (wt.%)	Sintered Density (g/cm^3^)	Porosity (%)	Relative Density (%)	ε_r_	d_33_ (pC/N)
PVC	0	7.10	12.56	87	1137	356
	5	6.98	14.04	85.96	346	231
	10	6.27	22.70	77.22	340	226
	15	5.94	26.85	73.15	311	213
SA	5	6.54	19.46	80.54	336	230
	10	6.01	25.98	74.01	264	206
	15	5.19	36.08	69.92	256	185
PMMA	1	5.06	37.68	63	356	236
	3	4.83	40.52	59.48	320	231
	5	4.42	45.57	54.43	182	228

## Abbreviations

ABS	Acrylonitrile butadiene styrene	PE	Polyethylene
AM	Additive manufacturing	PEMA	Poly(ethyl methacrylate)
BJ	Binder jetting	PFA	Pore forming agent
BT	Barium titanate	PMMA	Polymethyl methacrylate
BURPS	Burned out polymer spheres	PP	Polypropylene
DLP	Digital light processing	PSD	Particle size distribution
FDC	Fused deposition of ceramics	PVC	Polyvinyl chloride
FDM	Fused deposition modeling	PZT	Lead zirconate titanate
FGMs	Functionally graded materials	PZT-PCN	Lead zirconate titanate-lead cobalt niobate
FGP	Functionally graded piezoceramic	SA	Stearic acid
FOM	Figure of merit	SEM	Scanning electron microscope
HA	Hydroxyapatite	TPMSs	Triply periodic minimum surfaces
PDMS	Polydimethylsiloxane		
